# LEDs: Sources and Intrinsically Bandwidth-Limited Detectors

**DOI:** 10.3390/s17071673

**Published:** 2017-07-20

**Authors:** Roberto Filippo, Emanuele Taralli, Mauro Rajteri

**Affiliations:** 1 Nanoscience and materials division, INRiM—Istituto Nazionale di Ricerca Metrologica, Strada Delle Cacce, 91-10135 Torino TO, Italy; m.rajteri@inrim.it; 2 SRON—Netherlands Institute for Space Research, Sorbonnelaan 2, 3584 CA Utrecht, The Netherlands; e.taralli@sron.nl

**Keywords:** LED, light emitting diode, photodetector, radiometer, LED detector

## Abstract

The increasing demand for light emitting diodes (LEDs) is driven by a number of application categories, including display backlighting, communications, signage, and general illumination. Nowadays, they have also become attractive candidates as new photometric standards. In recent years, LEDs have started to be applied as wavelength-selective photo-detectors as well. Nevertheless, manufacturers’ datasheets are limited about LEDs used as sources in terms of degradation with operating time (aging) or shifting of the emission spectrum as a function of the forward current. On the contrary, as far as detection is concerned, information about spectral responsivity of LEDs is missing. We investigated, mainly from a radiometric point of view, more than 50 commercial LEDs of a wide variety of wavelength bands, ranging from ultraviolet (UV) to near infrared (NIR). Originally, the final aim was to find which LEDs could better work together as detector-emitter pairs for the creation of self-calibrating ground-viewing LED radiometers; however, the findings that we are sharing here following, have a general validity that could be exploited in several sensing applications.

## 1. Introduction

Light emitting diode (LED) development started in the early 1960s with the observation of infrared and red radiation emission, reaching blue wavelengths in the early 1990s [1] thanks to Akasaki, Amano, and Nakamura (Nobel prize winners, 2014), and is continuing deeper into the ultraviolet (UV). The extended wavelength range and the improved efficiency have allowed LEDs [2] to become the preferred light sources for many important applications, e.g., light sources in traffic signals [3], solid-state information and image displays [4], full-color illumination for back-lighting liquid crystal displays [5], automotive signaling and tail lights [6], instrument cluster displays [7], food production [8], analytical chemistry [9,10] microfluidics control [11] and, soon, in metrology as promising new photometric standards [12].

In all of these applications, LEDs are used as sources, but, although this is a more exploited LED property, it has been demonstrated that LEDs could also be applied as radiation sensors with an intrinsic bandwidth-limited sensitivity related to the emission spectrum [13,14,15]. This property is particularly interesting because it allows them to be selective in bandwidth without any dispersive or absorbing element, with great advantages from the physical dimension and cost point of view. There are already applications in radiometry in which LEDs have been used as bandwidth-limited photo-detectors: sun photometry [15,16], field radiometry [17,18,19], visible light communications [20], luminescence [21], and others. Unfortunately, these applications are still very limited and the companies producing LEDs do not provide any information on their devices’ detection properties. This knowledge is crucial to open new application fields.

In this paper we report the static radiometric behavior of more than 50 different models of LED, with the aim of: (a) starting to fill incomplete data regarding the behavior of LEDs used as sources (e.g., spectral emission and lambda peak dependence from forward current—*I_F_*—and aging reliability); and (b) providing a considerable set of data regarding the detection spectra of LEDs (that are usually related to their emission spectra, but never fully overlap [22]).

After some hints about the devices under test (DUTs) and their identification, in Section 2 we describe the measurement methods. The LED’s source properties are then reported in Section 3 while, in Section 4, the detection properties are summarized. Moreover, in Section 5 the matching between the source and detection spectra is considered, with a practical example that refers to the realization of our LED radiometer [18,19]. Finally, in Section 6, we summarize the main findings. Despite the fact that we investigated only the aspects of interest for our application, we believe that the knowledge of the technical details exposed in this article could be of help to the sensors community in the realization of low-cost detectors or matched source-detector couples in various applications.

## 2. Devices under Test and Experimental Setups 

The LEDs reviewed in this paper range from low to high power devices and cover the electromagnetic spectrum from 350 nm to 900 nm.

**Figure 1:**
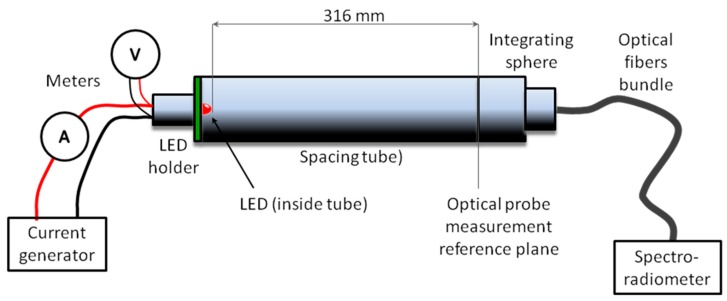
Experimental measurement diagram for emission parameters.

The DUTs were bought in several tranches. Upon delivery, we gave an identification number to each part number. Therefore, measurement data were labelled with letter “E” for “emission” measures or “D” for “detection” measures, followed by the identification number. All the measured parts are listed in Table 1, together with their id number. We also used these identifiers for the most crowded figure. Emission spectra have been acquired by means of a CAS 120 Array spectrophotometer (Instrument Systems GmbH, Munich, Germany) connected through an optical fiber bundle to a 25 mm diameter integrating sphere with a 100 mm^2^ acceptance aperture in combination with a spacing tube, so that the emitting device is placed at 316 mm from the reference plane of the optical probe (Figure 1). This system allows the measurement of the LED averaged radiant intensity, and other radiometric parameters, in Commission Internationale de l´Eclairage (CIE) condition A [23].

**Figure 2:**
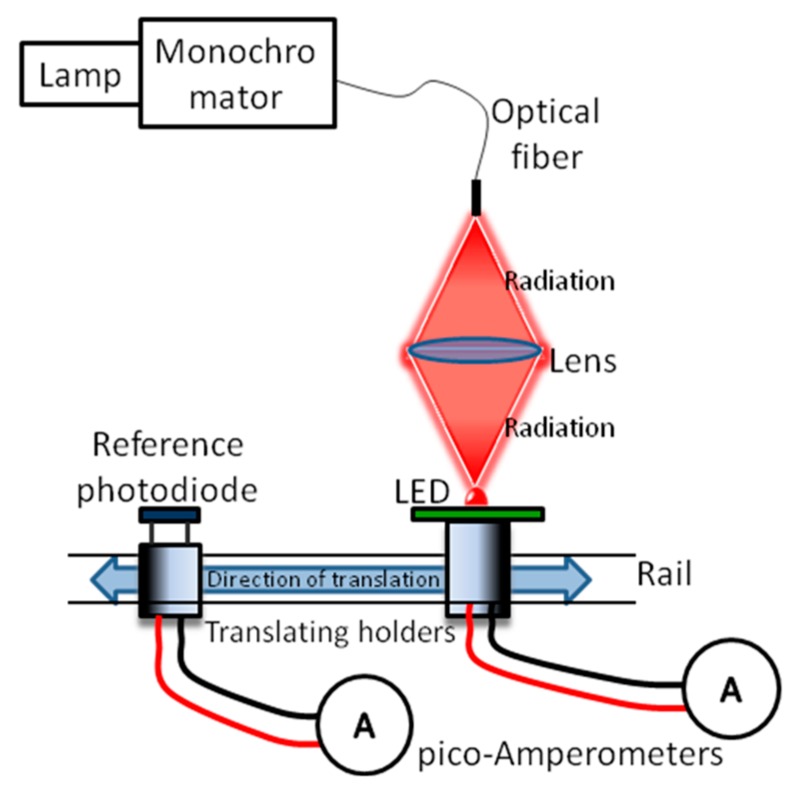
Experimental setup for the detection parameters measurement.

The spectral response of LEDs used as radiation sensors was obtained by placing each LED at the focal distance of a lens and irradiating through a fiber-optic connected to the exit of a monochromator, and measuring its correspondent output photocurrent by means of an electrometer (Figure 2). Since the spot size cannot be easily adjusted, the irradiance and the LED distance from the source are constant, however, the active area of the semiconductor might be equally-, over-, or under-filled, depending on its dimension. The photocurrent was compared with the photocurrent of a calibrated photodiode (used as reference) irradiated with the same flux of about 0.8 µW over the full spectrum with a tolerance of ±7.5%, coming from the same optical system. Finally, the spectral response of the LED detector under test *R_LED_*(*λ*) was calculated as:

(1) $$R_{LED}\left(\lambda \right)=R_{REF}\left(\lambda \right)\frac{I_{LED}\left(\lambda \right)-I_{LED0}\left(\lambda \right)}{I_{REF}\left(\lambda \right)-I_{REF0}\left(\lambda \right)}$$

where *R_REF_*(*λ*) is the calibrated spectral responsivity in A/W of the reference photodiode, *I_LED_*(*λ*) is the LED photocurrent under irradiation, and *I_LED_*_0_(*λ*) is the photocurrent caused by the stray radiation and the background noise, respectively; similarly, *I_REF_*(*λ*) and *I_REF_*_0_(*λ*) are the photocurrent under irradiation and the photocurrent caused by the stray radiation and the background noise, respectively, for the reference photodiode.

Since our application was very low speed, we did not study the dynamic behavior of the DUTs, such as the switching properties of the radiation emitters and the response time of the detectors. Furthermore, for noise considerations, we were not interested to the increase of the responsivity through the application of a reverse bias. However, both these aspects were discussed in [20].

## 3. LEDs as Radiation Sources 

**Figure 3:**
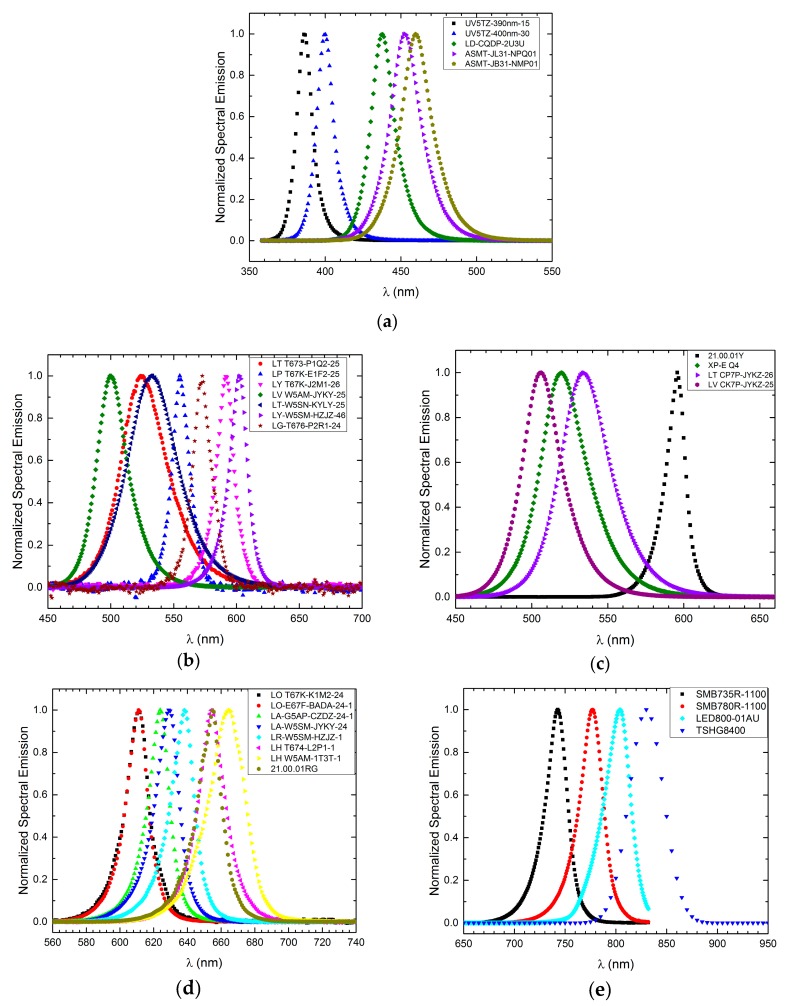
Examples from Table 1 of normalized emission spectra of LED sources with lambda peak ranging from: (**a**) 350 nm to 490 nm; (**b**) 490 nm to 650 nm (first group); (**c**) 490 nm to 650 nm (second group); (**d**) 600 nm to 700 nm; and (**e**) 700 nm to 830 nm.

In a radiation sensor, often a matched source of radiation is also needed to excite a physical phenomenon (as, for example, in [21]), with reference purpose (as in [18]), or for other use [24,25]. For this reason, we examined the emission properties of many LEDs. The measurement results are reported in Table 1 in the columns grouped under the “AS SOURCE” label. Several LED sources’ emission spectra are reported in Figure 3 and have been divided in four ranges: (a) UV-blue (peak wavelength range 350–490 nm), (b,c) blue-green (490–650 nm), (d) yellow-red (600–700 nm), concluding with NIR in (e) (700–830 nm). In each plot, the emission spectra are reported normalized to the maximum value.

The averaged radiant intensity of LEDs measured in CIE condition A [23], peak wavelength (*λ_PEAK_*) and bandwidth at full width at half maximum (Δ*λ_FWHM_*) are reported in Table 1.

Since energy consumption plays a key role in battery-supplied instrumentation, LED sources have been tested at current levels *I_F_* that, in certain cases, could be different from the nominal current and then, again, at a current *I’_F_* changed (reduced) by the percentage Δ*I_F_*, where:

(2) $$\Delta I_{F}=\left(\frac{{I^{\prime }}_{F}-I_{F}}{I_{F}}\right)\cdot 100$$

The resulting changes in terms of whatever quantity, i.e., *Radiant intensity* (Δ*Rad.Int.* vs. Δ*I_F_*), peak wavelength (Δ*λ_PEAK_* vs. Δ*I_F_*), and bandwidth (Δ*λ_FWHM_* vs. Δ*I_F_*), are expressed as percentages in the same table:

(3) $$(\Delta quantity\;\mathrm{vs}.\;\Delta I_{F})=\left(\frac{quantity({I^{\prime }}_{F})-quantity(I_{F})}{quantity(I_{F})}\right)\cdot 100.$$

Reducing the forward current to a percentage of the initial test value between 75% and 87.5%, depending on the LED, we observe a considerable reduction of Δ*λ_FWHM_* and *Radiant intensity* in the considered spectrum, while the reduction in *λ_PEAK_* is very limited. For a further decrease of the current, the emission spectra become too weak for a meaningful measurement. Due to the limitation of the spectral range of the spectrophotometer (360–830 nm), only three NIR LEDs were measured while the other NIR LEDs were tested only as detectors.

## 4. LEDs as Radiation Detectors

**Figure 4:**
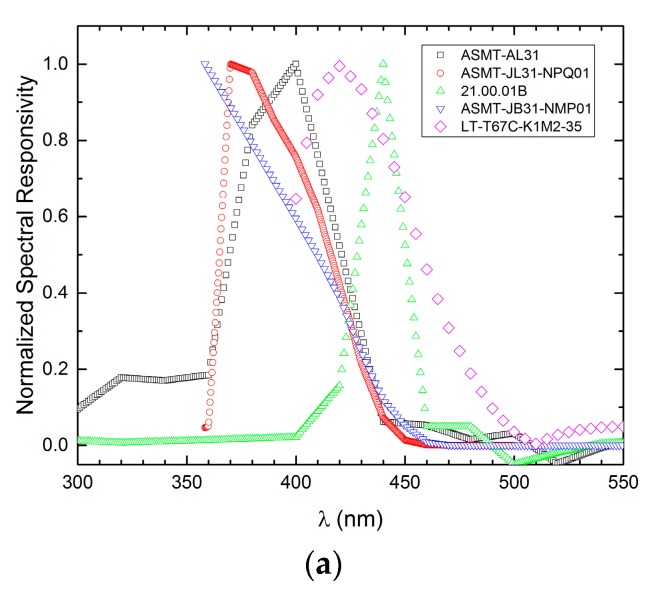

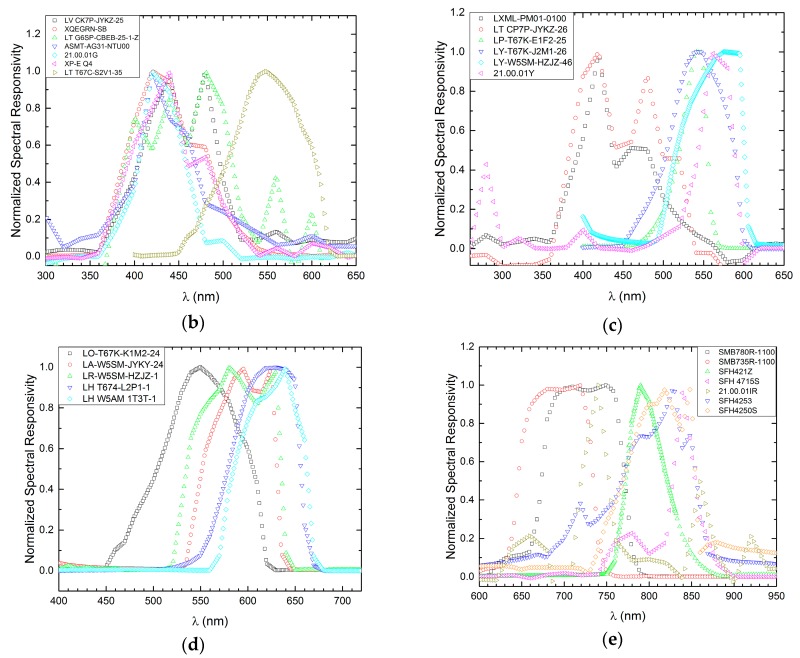
Normalized spectral detection of LED detectors in the peak wavelength range from: (**a**) 350 nm to 450 nm; (**b**) 400 nm to 600 nm (first group); (**c**) 400 nm to 600 nm (second group); (**d**) 550 nm to 650 nm; and (**e**) 650 nm to 850 nm.

The LED detection spectra in Figure 4 have been divided in four peak wavelength ranges:(a) 350–450 nm, (b) and (c) 400–600 nm (split in two groups), (d) 550–650 nm and, finally, (e) 650–850 nm.

Normally, the emission and detection spectra are shifted with respect to each other. The spectral bandwidth of the detection spectra goes from 20 to 120 nm and the the spectra themselves are generally asymmetric about the bandwidth center. All data are summarized in the last three columns of Table 1 where the *Responsivity index* is defined as:

(4) $$Responsivity\;index=\int_{FWHM}R_{LED}\left(\lambda \right)d\lambda .$$

The highest *Responsivity indices* are obtainable with power LEDs mainly due to the large collecting area since, as already mentioned, we could not make the radiation spot so small to under-fill the active area of the smaller LEDs. Additionally, because of the presence of domes or gel on the top of certain devices, the *Responsivity index* mainly contains two elements: the sensitivity of the DUT itself and the amount of captured energy due to the LED size with respect to the total available energy. This aspect must be considered when choosing LEDs for sensing, but has a negligible impact in several applications.

## 5. Matching of Source and Detector LEDs

**Figure 5:**
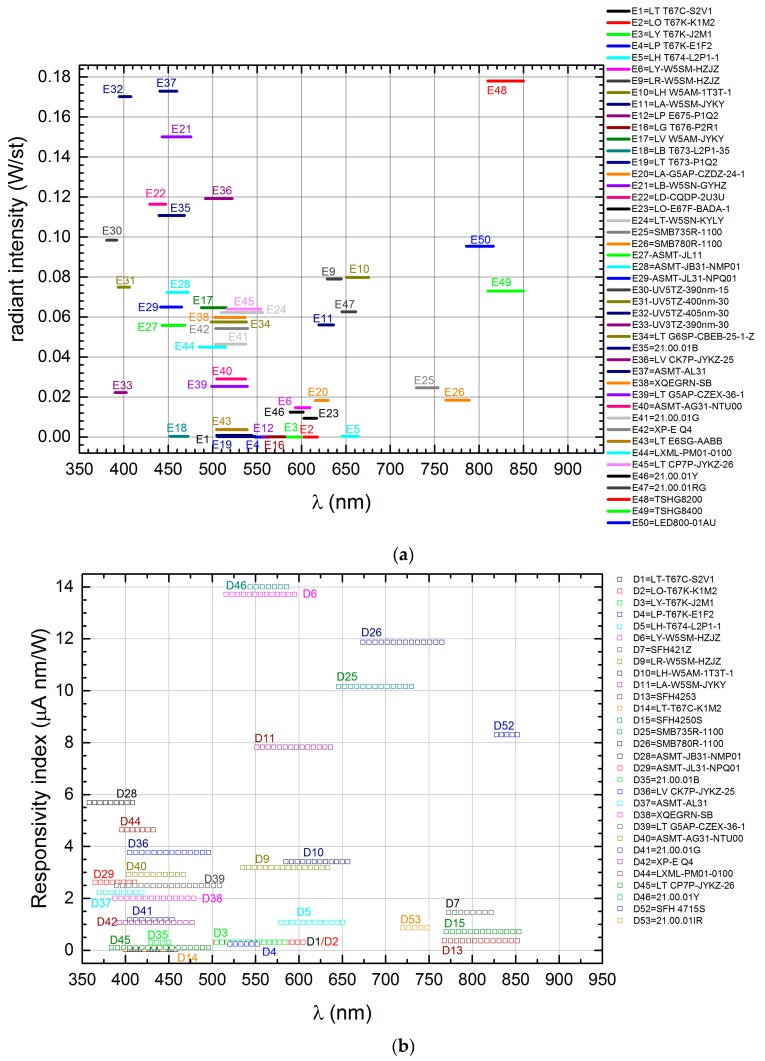
Representation of: (**a**) the *Radiant intensity* of LEDs used as radiation sources, and (**b**) the *Responsivity index* of LEDs used as radiation sensors. In both diagrams, the length of the segments corresponds to the FWHM, while in (**b**) the numerical value is the integral of the photocurrent over the FWHM.

The large amount of collected data, graphically represented in Figure 5, could be used to seek LEDs emitting in the desired bands with averaged radiant intensities that meet certain specifications, to find an off-the-shelf wavelength-selective detector, or to match source-detector devices on the required bands with an acceptable efficiency. Let us assume that we want to match a source and a detector in the range of blue-violet (that is, around 450 nm). From Figure 5a, we observe that some suitable source candidates are E37, E21, E35, and E29 while, from Figure 5b, we see that D36, D40, D39, D38, D42, and D45 are possible matching detectors.

Normally, we would choose E37 and D36 because of their, respectively, high *Radiant intensity* and high *Responsivity index*, however, further considerations might lead to other choices. For example, smoothness of the spectral responsivity curve might concern, or the possibility of trimming the peak emission wavelength, reducing the forward current, other energy considerations, reliability over the long-term, temperature effects, angular and temporal response, etc. 

**Figure 6:**
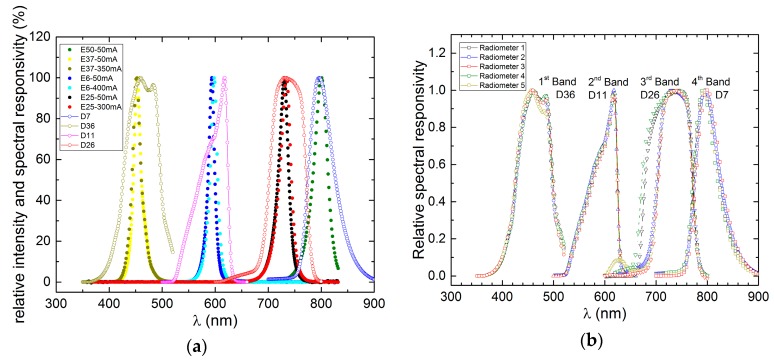
Normalized emission and detection spectra of the selected LEDs for: (**a**) one of the final radiometers; and (**b**) all five of the radiometers. Each radiometer mounts LED samples of the same family and they show good repeatability in terms of spectrum shape. The narrowing in the third band is due to the presence of a dome on top of the LED detector mounted in three of the radiometers.

As a practical example, Figure 6 shows four matched source-detector LED couples that were chosen for our realization of a self-calibrating radiometer based on LEDs used both as radiation-detecting devices and reference sources for the calibration [18,19].

Their relevant detection and emission spectra are shown in Figure 6a in the case of one radiometer. The choice is based of course on wavelength matching, but also on other important requirements: (i) to maximize the signal to noise ratio, the sources present high radiant intensities and the detectors, high R*esponsivity indices*; (ii) because of thermal considerations, the forward current of the sources is as low as possible, and (iii) for long-term stability, the degradation of the emitting LEDs as a function of operating time, is acceptably low. In order to meet requirement (ii), the use of sources with λ_PEAK_ and Δ*λ_FWHM_* that are as independent as possible from the forward current is crucial to reducing energy consumption. In Figure 6b, we report the normalized spectral *Responsivity index* of the LED detectors for all of the five radiometers that were built as final release. Each radiometer mounts LEDs of the same family that show good repeatability in terms of spectrum shape. The narrowing in the third band is due to the presence of a dome on top of the LED detector mounted in only three of the radiometers.

**Figure 7:**
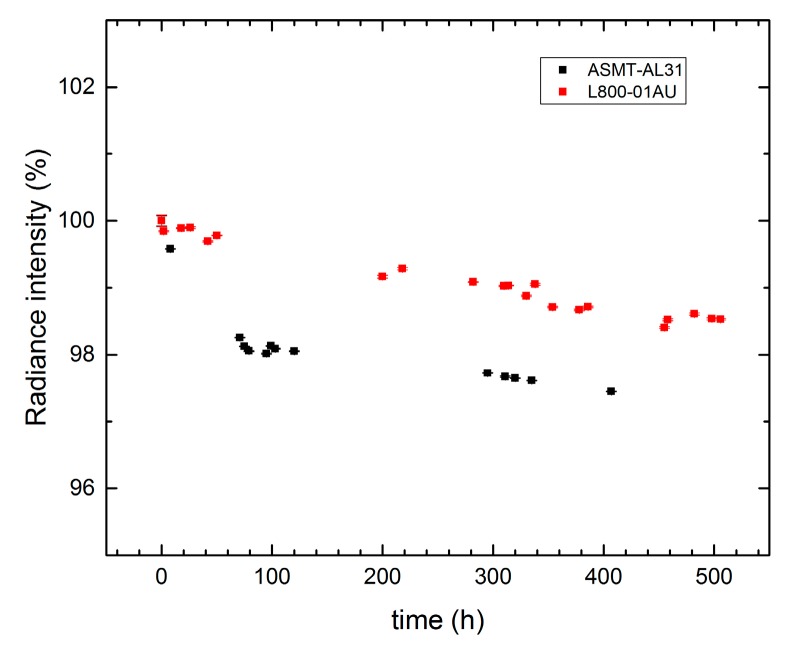
Radiance intensity as a function of the operating time in hours of the LED sources normalized to the initial value.

The LED sources were aged, firstly for the purpose of stabilization, and secondly to estimate possible physical change with time. LED *Radiant intensity* as a function of the operating time, normalized to the first measurement value (0 h), is shown in Figure 7 for two of the source LEDs. E37 (ASMT-AL31) of the first band shows an emission reduction about 2% within 100 h and a slight decrease of 0.5% in the remaining 300 h. LED source E50 (L800-01AU) of fourth band shows an emission reduction less than 2% over the 500 h; in particular, the reduction remain around 0.5% for the last 200 h. As shown, LED’s aging is an aspect not to be undervalued when a sensor needs a radiation exciter or reference.

## 6. Conclusions

The increasing applications of LEDs in photometry and radiometry, even as detectors, in addition to sources, has led to the study of various parameters of LEDs, which are relevant to radiometric measurements. The behavior of more than 50 LEDs was studied as sources and several of them as detectors, with the aim to begin filling the lack of data in manufacturers’ datasheets. It was observed experimentally that emission and detection spectra of the same LED are dissimilar in that the absolute peak wavelength of the emission spectrum is longer than the highest peak wavelength of the spectral response. The bandwidth of the two spectra are dissimilar and, in particular, in the case of LEDs used as detectors, it is asymmetric with respect to the peak wavelength. Moreover, LEDs of the same family show the same spectral response in terms of peak wavelength and bandwidth, but slight differences in the responsivity.

When decreasing the forward current of the LED sources, with the aim to limit the general energy consumption, we observed a slight reduction in the peak wavelength, while a reduction in the bandwidth, acceptable in most cases, was observed especially for LEDs in NIR spectral range. In addition, as one could expect, the *Radiant intensity* decreases proportionally to the reduction of the forward current. The data presented could be used as a starting point to design inexpensive wavelength-selective detectors or matching source-detector pairs of LEDs, which will save time; however, further investigations, especially regarding the reliability over the long-term, are suggested in the peculiar application to proof the suitability of the chosen LEDs to the sensor it will be used for.

## Figures and Tables

**Table 1:** List of LEDs characterized as sources and as detectors. The table is ordered with increasing emitted *λ_PEAK_*. LED columns legend: “P/N” is the commercial part number of the device under test; “Id” is the identification number that we gave to the part number in our tests, (in the following, “E” stands for emitting properties (reported in “AS SOURCE” columns) and “D” stands for detection properties (“AS DETECTOR” columns)). AS SOURCE columns legend: “*I_F_*” is the forward test current; “*λ_PEAK_*” is the wavelength of maximum emission; “Δ*λ_FWHM_*” is the emission bandwidth at full width at half maximum; “*Radiant intensity*” is the averaged LED radiant intensity measured with CIE method A; “Δ*λ_PEAK_*”, “Δ*λ_FWHM_*” and “Δ*Rad.Int.* vs. Δ*I_F_*” are the percentage variation of, *λ_PEAK_*, *λ_FWHM_* and *Radiant intensity*, respectively, measured when *I_F_* is changed according to “Δ*I_F_*”. AS DETECTOR columns legend: *λ_PEAK_* is the wavelength of maximum sensitivity of the LED, Δ*λ_FWHM_* is the sensitivity bandwidth at full width at half maximum; “*Responsivity index*” is calculated by applying Equation (1) and then Equation (4) (see text).

LED		AS SOURCE									AS DETECTOR	
P/N	Id	*I_F_* (mA)	*λ_PEAK_* (nm)	∆*λ_FWHM_*(nm)	*Radiant Intensity* (W/sr)	∆*I_F_* (%)	∆*λ_PEAK_* vs. ∆*I_F_* (%)	∆*λ_FWHM_* vs. ∆*I_F_* (%)	∆*Rad.Int.* vs. ∆*I_F_* (%)	*λ_PEAK_* (nm)	∆*λ _FWHM_* (nm)	*Responsivity Index* (μA nm/W)
UV5TZ-390-15	30	15	387.6	12.4	0.098	−75	−0.16	−5.6	−78.4	/	/	/
UV3TZ-390-30	33	15	396.6	13.0	0.022	−75	−1.7 × 10^-4^	−0.05	−70	/	/	/
UV5TZ-400-30	31	30	399.8	12.0	0.075	−75	−1.7 × 10^-4^	−0.07	−68	/	/	/
UV5TZ-405-30	32	30	401.6	13.0	0.170	/	/	/	/	/	/	/
LD-CQDP-2U3U	22	350	437.7	18.4	0.117	/	/	/	/	/	/	/
ASMT-AL31	37	350	450.0	19.5	0.173	−85	−0.45	−20	−83	400.0	51	2.23
ASMT-JL-31-NPQ01	29	350	452.6	24.0	0.059	−85	−0.02	−20	−82.5	370.5	51	2.62
21.00.01B	35	350	454.7	28.0	0.111	−85	−1.66	−17.2	−81.8	440.0	21	0.30
ASMT-JL11-NM	27	350	456.5	25.7	0.056	/	/	/	/	/	/	/
ASMT-JB-31-NMP01	28	350	459.9	24.0	0.065	/	/	/	/	358.3	52	5.69
LB-W5SN-GYHZ-25	21	700	460.0	32.6	/	/	/	/	/	/	/	/
LB-T673-L2P1-35	18	700	462.2	20.0	/	/	/	/	/	/	/	/
LT-T67C-K1M2-35	14	20	465.0	/	/	/	/	/	/	420.0	57	0.03
LV-W5AM-JYKY-25	17	350	501.7	28.0	0.065	/	/	/	/	/	/	/
LV CK7P-JYKZ-25	36	350	505.8	30.0	0.012	−85	−0.45	−17.2	−79	480.0	91	3.75
LT G6SP-CBEB-25-1-Z	34	350	516.4	39.8	0.057	−85	−0.06	−24	−71.6	/	/	/
XQEGRN-SB	38	350	517.6	34.0	0.060	−85	−1	−8.5	−80	439.0	97	2.00
LT G5AP-CZEX-36-1	39	350	518.0	40.4	0.025	−85	−0.05	23.4	−71.6	479.0	120	2.48
ASMT-AG31-NTU00	40	350	519.1	33.6	0.029	−85	−1.45	−10.6	−82.2	421.0	64	2.91
21.00.01G	41	350	519.7	34.0	0.046	−85	−1.3	−11.75	−78.6	423.0	53	1.18
XP-E Q4	42	350	519.7	37.0	0.054	−85	−1.2	−6.6	−65.2	439.0	89	1.06
LT-E6SG-AABB-36	43	30	519.9	34.7	0.004	/	/	/	/	/	/	/
LT-T67C-S2V1-35	1	20	522.3	40.0	0.0007	/	/	/	/	548.0	100	0.31
LT-T673-P1Q2-25	19	10	526.4	43.0	0.0002	/	/	/	/	/	/	/
LT-W5SN-KYLY-25	24	700	530.6	47.8	0.062	−85	−1	−27.3	−70.7	/	/	/
LXML-PM01-0100	44	350	530.7	39.0	0.045	−85	−0.65	15	−78.7	421.0	40	4.63
LT CP7P-JYKZ-26	45	350	533.9	39.0	0.064	−85	−0.92	−12	−81	418.0	114	0.10
LP-T67K-E1F2-25	4	2	555.2	16.0	8 × 10^−6^	/	/	/	/	544.0	35	0.24
LP-E675-P1Q2-25	12	50	563.4	14.0	8 × 10^−5^	/	/	/	/	/	/	/
LG-T676-P2R1-24	16	20	572.6	17.5	1 × 10^−4^	/	/	/	/	/	/	/
LY-T67K-J2M1-26	3	2	592.0	16.0	2 × 10^−5^	/	/	/	/	547.0	53	0.30
21.00.01Y	46	350	595.7	15	0.012	−85	−0.62	−13.3	−80.7	562	44	14.00
LY-W5SM-HZJZ-46	6	400	604	17.3	0.0146	90	1.5	18.7	76.3	575	84	13.70
LO-E67F-BADA-24-1	23	50	611.4	15.1	0.009	/	/	/	/	/	/	/
LO-T67K-K1M2-24	2	200	613.2	17.9	3 × 10^−5^	/	/	/	/	549.7	99	0.31
LA-G5AP-CZDZ-24-1	20	100	623.4	14.7	0.018	/	/	/	/	/	/	/
LA-W5SM-JYKY-24	11	400	628.6	16.5	0.056	−85	−1	18.6	−86.4	625.2	89	7.82
LR-W5SM-HZJZ-1	9	400	633.7	16.0	0.079	−85	−2	−21.7	−62	580.0	100	3.17
LH-T674-L2P1-1	5	10	654.3	19.0	3 × 10^−4^	/	/	/	/	629.7	75	1.07
21.00.01RG	47	350	655.1	16.0	0.062	−85	−0.9	−12.5	−85	/	/	/
LH-W5AM-1T3T-1	10	400	666.0	25.0	0.080	/	/	/	/	639.0	87	3.40
SMB735R-1100	25	800	773.4	35.2	0.025	−87.5	−5.3	−36.6	−83.6	720.0	88	10.16
SMB780R-1100	26	800	798.2	33.8	0.018	−87.5	−4.1	−27.7	−81.2	750.0	105	11.86
LED800-01AU	50	50	800.9	29.0	0.095	−75	−1	−7	−89	/	/	/
TSHG8200	48	100	830.0	40.0	0.178	/	/	/	/	/	/	/
TSHG8400	49	100	830.0	40.0	0.073							
SFH421Z	7	100	880.0	80.0	/	/	/	/	/	789.0	48	1.46
SFH4253	13	70	860.0	30.0	/	/	/	/	/	827.0	87	0.36
SFH4250S	15	70	860.0	30.0	/	/	/	/	/	849.0	84	0.912
SFH 4715S	52	1000	860.0	30.0	/	/	/	/	/	840.0	26	8.30
21.00.01IR	53	350	850.0	/	/	/	/	/	/	740.0	32	0.86
